# Different modalities to manage rheumatoid arthritis: an A to Z story

**DOI:** 10.2144/fsoa-2023-0134

**Published:** 2024-05-20

**Authors:** Mohamed Ahmed Raslan, Sara Ahmed Raslan, Eslam Mansour Shehata, Amr Saad Mahmoud, Nagwa Ali Sabri, Khalid J Alzahrani, Fuad M Alzahrani, Ibrahim F Halawani, Vasco Azevedo, Kenneth Lundstrom, Debmalya Barh

**Affiliations:** 1 Drug Research Center, P.O. Box 11799, Cairo, Egypt; 2 Department of Obstetrics & Gynecology, Faculty of Medicine, Ain Shams University, P.O. Box 11591, Cairo, Egypt; 3 Department of Clinical Pharmacy, Faculty of Pharmacy, Ain Shams University, P.O. Box 11566, Cairo,, Egypt; 4 Department of Clinical Laboratories Sciences, College of Applied Medical Sciences, Taif University, P.O. Box 11099, Taif, 21944, Saudi Arabia; 5 Department of Genetics, Ecology & Evolution, Institute of Biological Sciences, Federal University of Minas Gerais, Belo Horizonte, 31270-901, Brazil; 6 PanTherapeutics, Route de Lavaux 49, CH1095, Lutry, Switzerland; 7 Institute of Integrative Omics & Applied Biotechnology (IIOAB), Nonakuri, Purba, Medinipur, 721172, India

**Keywords:** anti-inflammatory, antioxidants, nutrition, rheumatoid arthritis, traditional treatment

## Abstract

**Aim:** To investigate different approaches to RA treatment that might lead to greater efficacy and better safety profiles. **Methods:** The Search strategy was based on medical subject headings, and screening and selection were based on inclusion/exclusion criteria. **Results & discussion:** Early therapy is critical for disease control and loss of bodily function. The most promising outcomes came from the development of disease-modifying anti-rheumatic drugs. Different foods have anti-inflammatory and antioxidant qualities that protect against the development of rheumatoid arthritis (RA). Some dietary patterns and supplements have been shown to have potential protective benefits against RA. **Conclusion:** Improvement in the quality of life of RA patients requires a tailored management approach based on the current patient medical data.

## Plain language summary

Rheumatoid arthritis is a complex disease with an unclear origin that affects the joints. In this systematic review, we aimed to investigate different effective ways of treating rheumatoid arthritis. Study results indicate that rheumatoid arthritis treatment requires coordination between different healthcare teams. As much as we can, when we start disease treatment early, this will lead to a better disease cure. Different drugs showed promising results in the treatment of rheumatoid arthritis, but the most promising treatment results came from a group of medicinal agents called ‘disease-modifying anti-rheumatic drugs’. Different foods have anti-inflammatory and antioxidant effect and help in protection against rheumatoid arthritis, but others, such as red meat and salt, have the opposite effect. Some dietary patterns and supplements, such as the Mediterranean Diet, vitamin D and probiotics, have been shown to have potential protective benefits against rheumatoid arthritis. Improvement in the quality of patient life requires an individualized management roadmap based on current patient medical data.

## Abstract (plain language summary)

Rheumatoid arthritis (RA) care necessitates a multidisciplinary approach due to its complexity.

Early therapy is critical for disease control, prevention of joint degradation, and loss of bodily function.

Changes in gut flora and body composition are two indirect ways through which nutrition impacts the onset and course of RA.

Mediterranean Diet (MD), vitamin D, and probiotics have been shown to have potential protective benefits against RA.

Excessive red meat intake and a high total protein supply have been linked to an increased risk of inflammatory polyarthritis.

Different molecules and minerals may contribute positively to relieving RA symptoms, preventing disease progression, and enhancing the quality of life of patients.

Lifestyle choices such as sedentary behavior, poor or unhealthy diet, smoking, and alcohol consumption are all factors associated with an increased risk of acquiring comorbidities with RA.

Genetic screening in healthcare settings is important to provide patients with the most suitable therapeutic choice with minimal side effects and maximum clinical efficacy.

## Graphical abstract

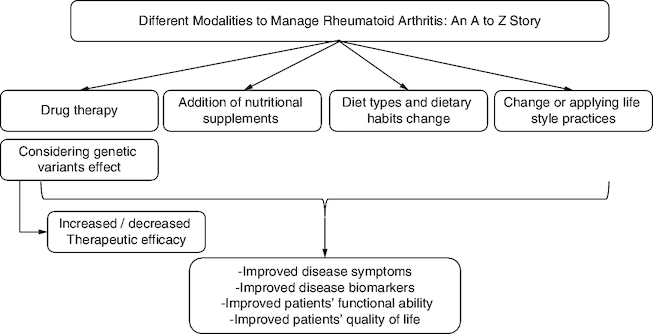

Rheumatoid arthritis (RA) is an autoimmune disorder that causes inflammatory arthritis as well as extra-articular manifestations and mostly affects synovial joints. It often begins in small peripheral joints, and it is commonly symmetric. If left untreated, it continues to affect proximal joints. Over time, joint inflammation causes cartilage and bone degradation, resulting in joint degeneration. Early RA is described as having symptoms for less than six months, while established RA is defined as having symptoms for more than 6 months [1]. Most clinical signs of RA show that the wrists, metacarpophalangeal joints, proximal interphalangeal joints and metatarsophalangeal joints in the foot are always painful and swollen [2].

The cause of RA is still unclear. It is hypothesized to be caused by the interplay of a patient's genetics and environment. Rheumatoid disease has a heritability of 40–65% for seropositive rheumatoid arthritis [3,4] and 20% for seronegative rheumatoid arthritis [5]. Genetic studies showed that *HLA-DRB1* alleles (HLA-DRB1*04, *01, and *10) have been linked to an increased chance of developing rheumatoid arthritis [6].

Cigarette smoking is the most significant environmental risk factor for rheumatoid arthritis. There is an association between genes and smoking that raises the incidence of RA in ACPA (anti-citrullinated protein antibody)-positive persons. On the other hand, the diversity of the gut microbiome changes in RA patients (dysbiosis) shows a lower gut microbiome diversity compared with healthy individuals. Collinsella has been linked to increased RA disease severity by altering the gut mucosal permeability [7].

RA generally appears between the ages of 35 and 60, with periods of remission and aggravation. It can also affect young children before the age of 16, which is known as juvenile RA (JRA), which is identical to RA except that no rheumatoid factor is present [8]. JRA is divided into five subtypes which are oligoarticular, polyarticular arthritis, systemic arthritis, enthesitis-related arthritis and psoriatic arthritis. The previous subtypes are seronegative except for polyarticular arthritis which may be seropositive like adults or seronegative [9]. The prevalence of RA in the West is estimated to be 1–2%, with a global incidence of 1% [8].

The interplay of genetic and environmental variables might cause RA in the potential trigger sites (lung, oral and gut), which is defined by the initiation of self-protein citrullination and the development of autoantibodies against citrullinated peptides [10].

In observational studies, diagnosed RA patients showed a poor nutritional status [11], with lower carbohydrate energy intake, higher saturated fat consumption [12], and lower micronutrient intake [11] compared with unaffected controls. This may contribute to the higher risk of cardiovascular diseases seen in RA patients [13].

Adequate antioxidant tissue concentrations may provide an essential defense against the increased oxidative stress in RA patients. Several studies have been conducted to investigate the benefits of vitamins C and E and selenium for the treatment of RA. In general, vitamin E (α-tocopherol) insufficiency and low tissue vitamin E levels boost inflammatory response components while suppressing immune response components [14].

New treatment techniques are now accessible as a result of significant breakthroughs in the pharmaceutical sector [15]. Early diagnosis and appropriate nonpharmacological and pharmacological treatment of RA, together with frequent monitoring of therapeutic efficacy and safety, are required for the most successful therapeutic strategy.

Treatment of RA aims to minimize joint inflammation and discomfort, improve joint function, and avoid joint deterioration and deformity. Treatment approaches include a mix of medications, weight-bearing exercise and patient education. Treatments are often tailored to the needs of the patient. It covers things like illness progression, the joints affected, age, general health, profession, compliance and disease education [16].

The objective of this systematic review is to present a collective summary of different pharmacological and non-pharmacological approaches for the management of RA based on comprehensive methodology as well as undertake a comparative evidence-based approach for different therapeutic and non-therapeutic modalities in order to provide a solution for the best therapeutic management for obtaining the best clinical outcome, increasing the quality of life of patients and minimizing potential side effects

## Methods

In the present review, the following sources were included: randomized controlled trials (RCTs), controlled non-randomized clinical trials (CCTs), retrospective and prospective comparative cohort studies, case control or nested case-control studies, reviews and systematic reviews. On the other hand, case reports and case series were excluded from the study sample.

**Figure 1.**
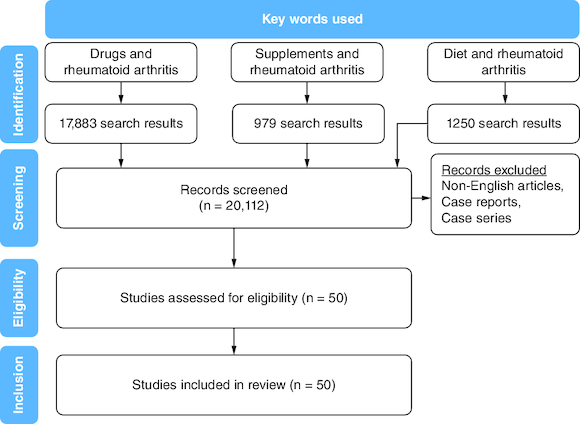
Flowchart of the article selection process.

A search strategy was constructed using medical subject headings (MeSH). The MeSH terms of supplements, nutrition, therapy, drugs, management, genetics and rheumatoid arthritis were used to systemically search PubMed and MEDLINE databases. Only studies in the English language with a study population of 18 years and older were included. All relevant publications up to 2022 were included. No limits regarding study design or date were set for the search. Duplicate studies were removed from our study pool (Figure 1). All included studies were scanned against inclusion and exclusion criteria. Our inclusion criteria (Table 1) primarily focused on published literature that assessed the effect of supplements, nutrition, drugs and genetic factors on RA management.

**Table 1.** Inclusion criteria for search strategy.

Parameter	Criterion
Participants	Human patients with RA
Intervention	Nutritional interventions, dietary supplements, therapeutics, fasting interventions and genetic factors
Comparator	Other dietary or therapeutic intervention, placebo supplement or no control group
Outcome	Laboratory and/or clinical and/or radiological and/or symptoms of RA and/or remission RA indices
Setting	All settings

The studies underwent review by two independent authors. In case there was a lack of agreement, a third author was sought for consultation, and the discrepancies were resolved through a collaborative discussion to achieve consensus.

All studies included underwent comprehensive data extraction. The extracted data encompassed various aspects, such as evaluation method, study duration, population included, interventions and outcomes related to the effect on rheumatoid arthritis risk and health-related quality of life.

## Results & discussion

### Different treatment modalities of rheumatoid arthritis

Because RA is an inflammatory disease, nonsteroidal anti-inflammatory drugs (NSAIDs) and glucocorticoids have typically been used as first-line treatments. They work quickly to alleviate RA pain and joint swelling.

**Figure 2.**
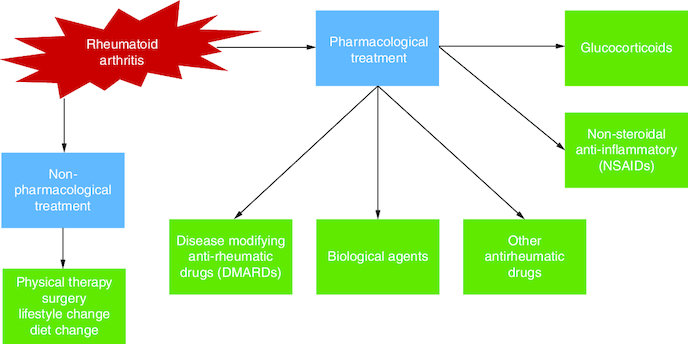
A diagram presenting the various treatment modalities for rheumatoid arthritis. The therapy options for RA are classified into two categories: non-pharmacological treatment and pharmacological treatment. Physical therapy, patient counseling on lifestyle variables, and surgical procedures to remove or replace the affected joint and bone regions are examples of non-pharmacological therapies. Pharmacological therapy includes NSAIDs, which are often used solely for symptomatic therapy or until a RA diagnosis is established since these therapies relieve pain and stiffness but have little effect on disease progression.

Methotrexate, hydroxychloroquine, sulfasalazine and, more recently, leflunomide are examples of disease-modifying antirheumatic medications (DMARDs). These slower-acting drugs, unlike NSAIDs, not only relieve symptoms but also decrease clinical and radiographic deterioration. Because their onset period spans from several weeks to months, more rapid-acting drugs, such as NSAIDs and glucocorticoids, are frequently used as ‘bridge’ treatments when starting DMARD therapy (Figure 2).

Biological-response modifiers, the most recent class of RA medicines, have been on the market for over 10 years. Examples include infliximab, etanercept, adalimumab, anakinra, abatacept and rituximab, which have been developed to target the inflammatory mediators of tissue destruction in RA (Table 2). Many additional therapeutic compounds are in various stages of clinical development and may be available in the next few years [2].

**Table 2.** Mechanisms of different categories of pharmacological therapeutics for the management of rheumatoid arthritis.

Category	Mechanism	Therapeutic agent	Ref.
NSAIDs	Inhibition of cyclooxygenase (COX)	Aspirin, ibuprofen	
Glucocorticoids	Inhibit cytokines and chemokines	Prednisone, methylprednisolone	
DMARDs	Reduction of neutrophil adhesion Inhibition of leukotriene B4 synthesis Reduction of levels of IL-6 and IL-8 Inhibition of TNF Suppression of IL-1 and TNF-α Preventing oxidative, nitrative, and nitrosative damage Inhibition of intracellular toll-like receptor TLR9	Methotrexate, hydroxychloroquine, sulfasalazine, and leflunomide	[17,18]
Other antirheumatic drugs	Inhibition of prostaglandin synthesis Inhibition of IL(s) Inhibits the expression of NOS	Gold sodium thiomalate, Cyclosporine, Tetracyclines	[19]
Biological agents	Anti-TNF-α IL-1 inhibitors Co-stimulation blockers B-cell-targeted therapies	Infliximab, Etanercept and Adalimumab Anakinra Abatacept Rituximab	

DMARD: Disease-modifying antirheumatic drug; NOS: Nitric oxide synthase; NSAID: Non-steroidal anti-inflammatory drug; TNF: Tumor necrosis factor.

Lung exposure to noxious substances, infectious agents including Porphyromonas gingivalis and Epstein-Barr virus (EBV), gut microbiota, and dietary variables may cause ACPA citrullination and maturation. Citrullination is mediated by the calcium-dependent enzyme peptidyl-arginine-deiminase (PAD), which results in a post-translational alteration of a positively charged arginine to a polar but neutral citrulline [10].

**Figure 3.**
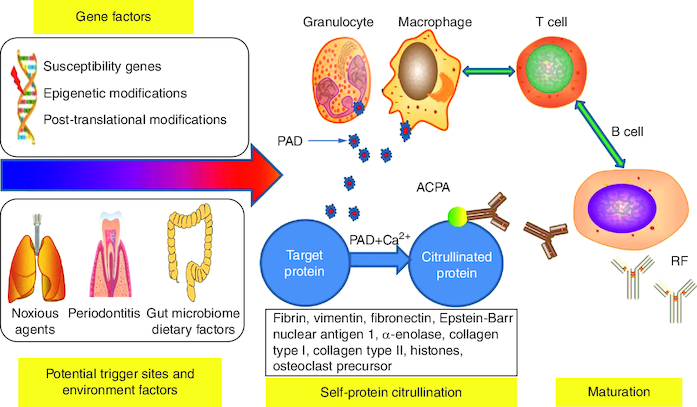
Triggering factors for rheumatoid arthritis. The mechanism and procedure for triggering action by the different factors can be explained as follows: **(A)** The interplay of genes and environmental variables at the various trigger sites, such as lung, mouth, and gut, might induce RA. **(B)** This triggering action is defined by the beginning of self-protein citrullination and the development of autoantibodies against citrullinated peptides. Data taken from [10].

PAD can be released by granulocytes and macrophages in RA (Figure 3). ACPA is caused by an aberrant antibody response to a variety of citrullinated proteins found throughout the body, including fibrin, fibronectin, EBNA1, type II collagen, and histones. Many citrullination neoantigens activate MHC class II-dependent T cells, assisting B cells in producing more ACPA [10].

Treatment success and failure are crucial in management of any disease condition. The adverse events of medication and disease conditions play a major role in the success or failure of RA treatment. Because of the danger of acquiring major infections and malignancies, a significant number of RA patients have refractory disease and treatment interruptions. Although interleukins (IL-1, IL-17A), and p38 play important roles in RA pathogenesis, various drugs targeting these variables have not been licensed due to their limited effectiveness and severe side effects (Tables 3 & 4).

**Table 3.** Summarized data of different referenced studies showing different treatment modalities with treatment failure of rheumatoid arthritis.

Category	Clinical outcome	Contribution factors to failure	Ref.
DMARDs	Treatment failure of Sulfasalazine (88.9%) MTX (75%) HCQ (72.2%)	Adverse effects reported (commonly bone marrow suppression and hepatotoxicity) for Sulfasalazine and MTX Addition or replacement of DMARDs for those on HCQ	[20]
Biological agents	58% overall failure for biological therapy (77% primary failure, and 23% secondary failure)	Patients with negative rheumatoid factor Patients using low-dose steroids Patients with a longer disease duration	[21]

DMARD: Disease-modifying antirheumatic drug, HCQ: Hydroxychloroquine; MTX: Methotrexate.

**Table 4.** Summarized data from studies of treatment success of rheumatoid arthritis.

Category/drug	Clinical outcome	Success mechanism	Ref.
Abatacept	Inhibition of the upstream immune synapse, including the APC-T-cell-B-cell axis, by abatacept can minimize compensatory pathway activity by inhibiting various downstream cytokines Lower incidence of serious infections in patients with RA, compared with other biologics	Targeting CD80/CD86	[22]
Baricitinib	Exhibited superior efficacy compared with MTX monotherapy	JAK/STAT inhibition	[23]

APC: Antigen presenting cell; CD: Cluster of differentiation; JAK: Janus kinases; RA: Rheumatoid arthritis; STAT: Signal transducer and activator of transcription.

Leading factors for treatment failure of rheumatoid arthritis include the incidence of adverse effects that may lead to treatment discontinuation or noncompliance. Also, lack of treatment efficacy and prolonged disease conditions that were not treated previously can lead to treatment failure.

Treatment success is dependent on the proper selection of the appropriate therapeutic drug, an appropriate dose and dosing intervals. Additionally, compliance with a healthy lifestyle, dietary control and early disease diagnosis and treatment are necessary.

### The effect of different nutrients, food sources, & therapeutic agents on the clinical outcome of rheumatoid arthritis

Different studies were analyzed for evaluation of different therapeutic approaches on the clinical outcome. A common finding across all studies analyzed was that improvements in RA disease activity were most noticeable for the majority of disease components (Table 5).

**Table 5.** Summarized data for referenced studies investigating the effect of different nutrients, food sources and drugs on RA risk and clinical outcomes.

Evaluation method	Duration	Population	Nutrients/foods/drugs	Outcomes (effect on RA risk)	Ref.
Self-administrated weekly retrospective FFQ	1 year (2012–2013)	968 RA patients 1037 healthy controls	Potatoes Fruits (no citrus fruits) Citrus fruits Mushrooms Dairy products Red meats Vegetables	Harmful effect No effects Protective effect Protective effect Protective effect No effects No effects	[24]
Prospective semi-quantitative FFQ at baseline and every 2 years in follow-up	22 years (1980–2002)	82,064 women in NHS (546 RA)	Proteins Iron Red meats Fish	No effects	[25]
Randomized, single-blind controlled trial. Evaluation done after 1 month of dietary regimen	3.5 months	27 RA patients on vegan diet, 26 RA Control patients	Gluten-free vegan diet followed by lactovegetarian diet	Significant decrease in leukocyte and platelet count IgM rheumatoid factors, IgG, C3 and C4 complement Calprotectin	[26]
Pilot study	2 weeks	21 RA patients on elemental diet, 9 RA patients on prednisolone 15 mg/day (Control)	An elemental diet contains amino acids (no whole proteins), mono/di-saccharides, medium/long-chain triglycerides supplemented with vitamins and trace elements	Significant improvement in Morning stiffness Ritchie articular index Clinical parameters were improved by 20% in 72% patients in elemental diet group as compared with 78% in steroid group	[27]
Randomized double-blind clinical trial	8 weeks	22 RA patients on probiotics, 24 RA patients (placebo)	10^(8)^ CFU of *L. casei* 01 supplement	Decreased CRP levels and counts of swollen and tender joints	[28]
Randomized, double-blind, placebo-controlled trial	8 weeks	30 RA patients on probiotics, 30 RA patients (placebo)	*Lactobacillus acidophilus* (2 × 10^(9)^ colony-forming units [CFU]/g), *Lactobacillus casei* (2 × 10^(9)^ CFU/g) and *Bifidobacterium bifidum* (2 × 10^(9)^ CFU/g)	Improved DAS28 Decrease in serum insulin levels and CRP levels	[29]
Food Challenge Trial	12 days	20 RA patients +ve SPT 20 RA patients -ve SPT	Allergenic food: dairy products, eggs, meat, fish, refined sugar, wheat, wheat flour, corn, rice, nuts.	72% patients in PPG group and 18% in PNG group suffered from disease aggravation on food challenge Increased levels of TNF-alpha, IL-1beta, ESR and CRP	[30]
Randomized, double-blind, placebo-controlled, parallel-design, clinical pilot	2 months	22 RA patients on probiotics, 22 RA patients (placebo)	Bacillus coagulans GBI-30, 6086	Reduction in total CRP Increase the ability to walk 2 miles, reach and participate in daily activities Pain improvement	[31]
Randomized, single-blinded, controlled crossover trial	10 weeks	50 RA patients (randomly assigned to an intervention diet or a control diet) 44 RA patients Competed both diet periods	Intervention diet: Fish (mainly salmon) three to four times per week, vegetarian dishes with legumes one to two times per week Potatoes, whole-grain cereals, vegetables, yoghurt for sauces, spices, and other flavorings were also included Snacks were composed of fruits Breakfasts contained low-fat dairy, pomegranate, whole-grain cereals, and nuts, blueberries and juice + probiotics Control diet Contained meat or chicken and refined grains daily, protein bar or quark for snacks Breakfasts based on either white bread with a butter-based spread and cheese, or a mix of quark and yoghurt with corn flakes and orange juice	Non-significant decrease in ESR in intervention group	[32]
Randomized, controlled trial	3 months	23 RA patients on Cranberry juice, 18 RA patients (Control)	Usual diet plus 500 ml/d of reduced calorie cranberry juice	Decreased disease activity Decreased DAS28 values Decreased anti-CCP values	[33]
Randomized Cross-Over trial	11 weeks	20 RA patients on blue mussels, 19 RA patients (Control)	Intervention meal: Five weekly meals with 75 g of blue mussels in addition to normal diet Control meal: Five weekly meals with meat/chicken in addition to normal diet	Reduced CRP, and disease symptoms Improved perceived health	[34]
Randomized double-blind placebo-controlled clinical trial	12 weeks	35 RA patients on ginger powder, 35 RA patients (placebo)	1500 mg ginger powder in two capsules daily	Reduction in disease activity score Increasing *FoxP3* genes expression Decreasing *RORγt* and *T-bet* genes expression	[35]
Randomized double-blind clinical trial	8 weeks	20 RA patients on cinnamon powder 20 RA patients (Placebo)	2000 mg cinnamon powder in four capsules daily	Significant decrease of serum levels of CRP and TNF-α Significant decrease in diastolic blood pressure Reduced swollen and tender joints score	[36]
Randomized controlled, double-blind trial	3 months	33 RA patients on saffron, 33 RA patients (Placebo)	100 mg/d saffron in one tablet	Decreased the number of tender and swollen joints Decreased pain intensity Decreased ESR, CRP, TNF-α, IFN-γ and malondialdehyde Increased total antioxidant capacity	[37]
Randomized controlled, unblinded trial	12 weeks	29 RA patients on a Mediterranean diet, 27 RA patients (control)	Mediterranean diet Some meals in the initial 3 weeks. Margarine, olive oil, canola oil, frozen vegetables, tea.	Reduction in inflammatory activity Increase in physical function Improved vitality Reduced pain and swollen joint count Decreased CRP	[38]
Randomized controlled, double-blind trial	8 weeks	35 RA patients on alpha lipoic acid, 35 RA patients (Control)	Two capsules of 1200 mg alpha lipoic acid	Serum inflammatory biomarkers and MMP-3 were not significantly affected No significant decrease in serum levels of CRP, TNF-α, IL-6	[39]
Randomized crossover, placebo-controlled, double-blind trial	10 weeks	38 RA patients on intervention, 38 RA patients (Control)	Intervention nutrients 8 g micro algae, enriched in 60 g sausage, 8 g tomato spread, 30 g milk powder Amount of n3/d: 2.36 g Control 8 g sunflower oil, enriched in 60 g sausage, 8 g tomato spread, 30 g milk powder	Reduction in clinical and biochemical signs of inflammation	[40]
Randomized controlled, double-blind trial	3 months	21 RA patients on vitamin D3, 18 RA patients (Control)	300 000 IU (7500 μg) of vitamin D_3_ administered once	Ameliorates the general health of patients	[41]
Randomized controlled, double-blind trial	12 months	11 RA patients on vitamin D2, 11 RA patients (Control)	Intervention Month 1: 3*50 000 IU vitamin D2/week Month 2–11: 2*50 000 IU vitamin D2/month, 1500 mg calcium daily Placebo 1500 mg calcium daily	No improvements in disease activity, or cytokines levels	[42]
Randomized controlled, double-blind trial	28 days	18 RA patients on intervention, 18 RA patients (Control)	Intervention 6000 mg of potassium in the form of enriched white grape juice Placebo grape juice	Decreased pain intensity	[43]
Randomized controlled, double-blind trial	2 months	27 RA patients on intervention, 27 RA patients (Control)	100 mg/day CoQ10 capsules	Reduction in ESR Reduction tender in joint count Reduction in pain score, and serum MMP-3 level	[44]
Randomized controlled, unblinded trial	3 months	50 RA patients on intervention, 50 RA patients (Control)	One capsule containing 1 g resveratrol daily	Decreased level of CRP, ESR, MMP-3, TNF-α, and IL-6 Lowered disease activity Decreased swelling and tenderness	[45]
Randomized controlled, double-blind trial	12 weeks	49 RA patients on intervention, 48 RA patients (Control)	10 g cod liver oil in capsules containing: 1500 mg EPA, 700 mg DHA, 800 μg vit A, 5 μg vit D, 20 IE vit E	No significant effect on RA disease activity	[46]
Randomized controlled, double-blind trial	8 weeks	32 RA patients on intervention, 32 RA patients (Control)	10 μg/day of vitamin K1 as a chewable tablet	No significant effects on blood inflammatory biomarkers No significant effect on RA disease severity	[47]
Randomized controlled, double-blind trial	8 weeks	25 RA patients on intervention, 25 RA patients (Control)	One capsule containing 500 mg quercetin	Reduced early morning stiffness Reduced morning pain, and after-activity pain	[48]
Randomized controlled, double-blind trial	8 weeks	27 RA patients on intervention, 27 RA patients (Control)	Synbiotic capsules containing *L. acidophilus*, *L. casei*, *B. bifidum* (each 2 × 10^9^ CFU) and 800 mg insulin	Reduced CRP Reduced disease activity Reduced pain	[49]
Randomized controlled, double-blind trial	3 months	15 RA patients on intervention, 14 RA patients (Control)	Two daily capsules of *L. rhamnosus* GR-1 and *L. reuteri* RC-14 (each 2 billion CFU), plus dextrose, potato starch, microcrystalline cellulose and magnesium stearate	No Clinical improvement Occurrence of functional improvement	[50]
Randomized, double-blind, placebo-controlled, parallel-design trial	8 weeks	31 RA patients on intervention, 31 RA patients (Control)	500 mg garlic tablets (equivalent to 2500 mg of fresh garlic, and containing 2.5 mg allicin) twice daily	Significant increment in serum TAC Significant reduction in MDA levels Improvement HAQ	[51]
A Randomized, Double-Blind, Placebo-Controlled, Two-Dose, Three-Arm, and Parallel-Group Study	12 weeks	12 RA patients on intervention 1, 12 RA patients on intervention 2, 12 RA patients (Control)	Intervention 1: curcumin 250 mg twice a day Intervention 2: curcumin 500 mg twice a day	Significant improvement in VAS, CRP, DAS28, ESR, and RF values in both interventions compared with placebo.	[52]
Randomized controlled trial	54 weeks	86 RA patients on intervention, 88 RA patients (Control)	Intervention: methotrexate + infliximab 3 mg/kg I.V every 4 or 8 weeks Control: methotrexate + placebo	Sustained reduction in the symptoms and signs of rheumatoid arthritis Quality of life significantly better	[53]
Randomized controlled trial	54 weeks	359 RA patients on intervention 1, 363 RA patients on intervention 2, 282 RA patients (Control)	Intervention 1: methotrexate + infliximab 3 mg/kg I.V Intervention 2: methotrexate + infliximab 6 mg/kg I.V Control: methotrexate + placebo	Significant improvement in physical function on intervention 1 and 2 Less radiographic progression on intervention 1 and 2	[54]
Randomized controlled trial	24 weeks	67 RA patients on intervention, 62 RA patients (Control)	Intervention: adalimumab 40 mg s.c. every 2 weeks + Methotrexate Control: methotrexate + placebo	Significant, rapid, and sustained improvement in disease activity	[55]
Randomized controlled trial	52 weeks	207 RA patients on intervention, 200 RA patients (Control)	Intervention: Adalimumab 40 mg s.c. every 2 weeks + Methotrexate Control: Methotrexate + placebo	Significant less radiographic progression Significant improvement of physical function	[56]
Multicenter, randomized, double-blind, placebo-controlled trial	24 weeks	59 RA patients on intervention, 74 RA patients (Control)	Intervention: anakinra 1 mg/kg/day s.c. + methotrexate Control: methotrexate + placebo	Significant improvements in the HAQ disability index	[57]
Randomized, double-blind, placebo-controlled study	6 months	115 RA patients on intervention, 119 RA patients (Control)	Intervention: abatacept 10 mg/kg i.v. + methotrexate Control: methotrexate + placebo	Significant improvement in disease signs and symptoms and health-related quality of life	[58]
Multicenter, randomized, double-blind, controlled study	48 weeks	40 RA patients on intervention, 40 RA patients (Control)	Intervention: rituximab 1 g i.v. + methotrexate on days 1 and 15 Control: methotrexate + placebo	Improvement in disease symptoms	[59]
Randomized, double-blind, placebo-controlled trial	6 months	78 RA patients on intervention, 80 RA patients (Control)	Intervention: Etanercept 25 mg s.c. twice weekly Control: olacebo	Significant reduction of disease activity	[60]
Multicenter, double-blind, placebo-controlled trial	3 months	55 RA patients on intervention, 53 RA patients (Control)	Intervention: tocilizumab 8 mg/kg every 4 weeks Control: placebo	Reduced disease activity	[61]
Double-blind, placebo-controlled	6 months	20 RA patients on intervention, 20 RA patients (Control)	Intervention: HCQ 200 mg and MTX (7.5 mg) once a week Control: HCQ (200 mg daily) and placebo	Reduced radiological progression in intervention group	[62]
Double-blind randomized trial	15 weeks	RA patients	Intervention: fish oil derived (n-3) fatty acid supplementation (three to six capsules/day) Control: olive/corn oil capsule supplement	Improvement HAQ, MS	[63]
Randomized, controlled, single blinded, study	6 months	33 RA patients on intervention, 33 RA patients (Control)	Intervention: Metformin 850 mg twice daily Control: placebo twice daily	Significant improved inflammation, disease severity, and quality of life Significant decrease in CRP levels	[64]

Anti-CCP: Anticyclic citrullinated peptide; CoQ10: Coenzyme Q10; CRP: C-reactive protein; DAS28: Disease Activity Score 28; EPIC-Norfolk: European Prospective Investigation of Cancer in Norfolk; ESR: Erythrocyte sedimentation rate; FFQ: Food frequency questionnaire; HAQ: Health Assessment Questionnaire; IL-6: Interleukin-6; IP: Inflammatory polyarthritis; MDA: Malondialdehyde; MMP-3: Matrix metalloproteinase-3; MMP-3: Serum matrix metalloproteinases; MS: Morning stiffness.; NHS: Nurses' Health Study; PNG: Prick-negative group; PPG: Prick-positive group; RA: Rheumatoid arthritis; SPT: Skin prick test; TAC: Total Antioxidant Capacity; VAS: Visual analog scale.

Dietary sources, which include a gluten-free diet, probiotics either as a supplement or from dietary sources, cranberry juice, ginger, and cinnamon, showed a significant clinical improvement in RA disease condition.

On the other hand, some studies that included vitamin D2, cod liver oil, alpha lipoic acid, and vitamin K1 supplementation reported no contribution of these nutrients to the improvement of RA disease activity or superior clinical outcomes.

**Figure 4.**
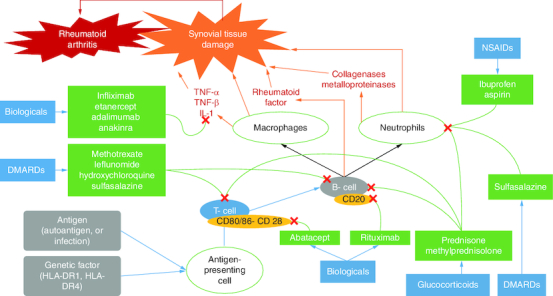
Different pathways for the therapeutic agents used in relieving the symptoms of rheumatoid arthritis. The pathways are different and can be summarized as follows: **(A)** Control of the immune cells and inflammatory mediators leads to suppression of the inflammatory response and reduction of the activity of the disease (RA). **(B)** Different molecules contribute to blocking T- and B-cell-mediated responses, like glucocorticoids, DMARDs and biological agents. **(C)** Biological agents contribute to inflammatory mediator inhibition. **(D)** NSAIDs inhibit neutrophil aggregation.

The current therapeutic agents used (Figure 4) to treat RA include pain and inflammation management agents such as NSAIDs. The first-line therapy for all newly diagnosed cases of RA includes DMARDs, biological-response modifiers, and targeted agents. Furthermore, glucocorticoids and other antirheumatic therapeutic agents are also used for RA treatment.

### Effect of dietary & lifestyle habits on rheumatoid arthritis

RA is associated with an increased risk of acquiring comorbidities, some of which are known to be associated with lifestyle choices such as sedentary behavior, poor or unhealthy diet, smoking, and alcohol consumption. According to several studies, individuals with RA do not engage in health-promoting physical activity. Obesity and diet are strongly related, and obesity is associated with disease severity and a greater number of comorbidities in RA patients [65].

**Figure 5.**
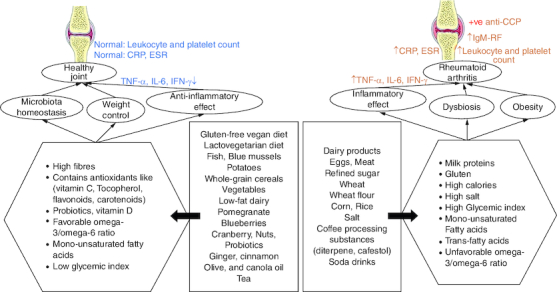
The indirect correlation between different dietary products and the progression of rheumatoid arthritis. **(A)** The effect depends on how food affects inflammatory responses, normal microbiota, and weight control. **(B)** Usually, diet rich in fiber, vitamins, omega 3 and low-glycemic index foods contribute to protection from RA incidence. **(C)** Diets with high salt, high calories and a high glycemic index contribute to RA occurrence or incidence.

**Figure 6.**
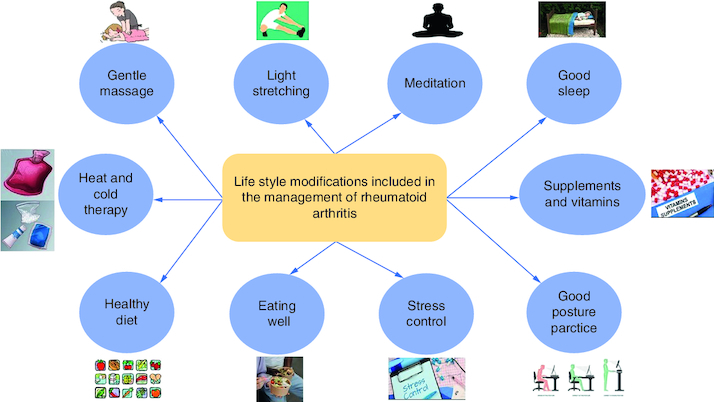
Several lifestyle practices that might be beneficial, when applied by the patients, in controlling and managing rheumatoid arthritis. **(A)** The contributing factors for enhancing RA management include dietary habits like healthy food, eating well (eating sufficient quantities of food to provide the required daily caloric need), and supplements. **(B)** Taking care of physical wellbeing includes good posture practice, regular light exercise, massage, heat, and cold therapy. **(C)** Emotional, psychological, and mental wellbeing include stress control, meditation, and good and sufficient sleep.

Based on the qualities of individual foods (Figure 5), dietary habits may constitute both a disease risk and a preventive factor. Specific foods can indeed show pro-inflammatory effects (for example, salt, red meat and high caloric intake) or, in contrast, reduce inflammation (oils, fatty fish, fruit and others). Furthermore, diets represent a major factor influencing microbiota composition, which is involved in disease development. Moreover, changes in lifestyle (Figure 6) can help with RA management.

**Figure 7.**
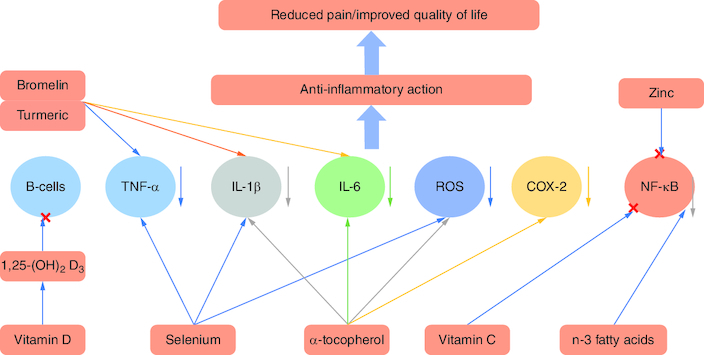
Effect of different molecules and minerals on rheumatoid arthritis. **(A)** Nutrients affect cells and mediators of the immune-inflammatory ‘cascade’ leading to RA. **(B)** Once the mediators or cells are controlled, the inflammatory response will be mitigated, causing improvements in the signs and symptoms of RA.

Different molecules and minerals may contribute positively to relieving RA symptoms, preventing disease progression, and enhancing the quality of life of patients. Some supplements suppress TNF-α, IL-1β and IL-6, such as selenium, α-tocopherol, bromelin and turmeric. Other molecules and minerals like vitamin C, n-3 fatty acids and zinc can contribute to the suppression of the NF-κB pathway. Vitamin D3 has been shown to mitigate B-cell function [66]. Reactive oxygen species are neutralized by antioxidant supplements like vitamin C and α-tocopherol [67]. The blockage of these pathways/inflammatory cytokines results in an anti-inflammatory effect and reduced joint pain (Figure 7).

### Exercise (light stretching), gentle massage & posture

The pain and stiffness of RA patients may discourage exercise, leading to a sedentary lifestyle, which can exacerbate RA-related symptoms. Failure of proper joint function might weaken the surrounding muscles. Tendons and soft tissue can become inflamed, which can eventually lead to increased joint instability.

Exercise on a regular basis gets the blood flowing into the muscles and can increase the synovial fluid, which can contribute to prevention or reversal of inflammation and joint stiffness. Massage can enhance blood flow through the muscles surrounding the joints, which can reduce joint swelling and relieve pain.

### Stress management, sleep wellness & meditation

Stress management can reduce pain and depression by promoting positive improvements in the areas of self-efficacy, coping strategies and overcoming helplessness regarding the control of RA [68]. According to a clinical trial, sleep disruptions are common in RA patients and may increase symptom severity [69]. Furthermore, a meta-analysis found that yoga may be effective for increased physical function, grip strength and reduced disease activity in RA patients [70].

### Heat & cold therapy

For individuals with RA, superficial heat and cold treatments are routinely employed [71]. Both methods provide analgesic relief. Temperature changes can affect blood flow, inflammation and nerve sensation. Heat and cold significantly increase the pain threshold immediately after application. Massage can help RA patients reduce suffering and the need for analgesics.

### Diet as a risk & protective factor for rheumatoid arthritis

Based on data that supports the unique qualities of different foods and their potential to increase or decrease inflammation, diet may be both a risk factor and a protective factor. Until now, research on this issue has produced contradictory results. Study designs and sample sizes have varied, which have also affected the interpretations.

Excessive red meat intake and a high total protein supply have been linked to an increased risk of inflammatory polyarthritis. Potential reasons for this link include increased inflammation caused by meat fats and nitrites, in addition to increased synovial involvement caused by an excessive oral iron load [72].

High dietary sodium (salt) consumption, which is typical in Western countries, has been linked to an increased risk of RA [73]. High salt levels may exacerbate the negative effects of other environmental variables, particularly smoking, by promoting SGK-1 expression, which leads to increased Th17 cell differentiation and autoimmune enhancement [74].

Clinical research has found that individuals with inflammatory arthritis consume fewer fruits and less vitamin C than controls, and that olive oil consumers have a lower chance of developing RA [75]. Fruits, vegetables, and olive oil may reduce the incidence of RA by supplying antioxidant elements, such as tocopherols found in olive oil, which serve as free radical scavengers [76].

It was found that drinking sugar-sweetened soda on a daily basis increases the risk of RA. High-fructose-flavored soft drinks may help promote arthritis development in young adults by producing an excessive buildup of glycation products that enhance inflammation [77].

Mikuls *et al.* observed a higher risk of seropositive rheumatoid in those who drank four or more cups of coffee per day, but people who drank more than three cups of tea per day had a lower risk [78]. Furthermore, Heliövaara *et al.* reported a positive relationship between coffee intake and rheumatoid factor (RF) positivity [79].

Diallyl sulphide, a garlic aroma component, has been shown to suppress mediated NF-κB and MAPK signaling pathways in murine macrophage-like cells. It also reduced Porphyromonas gingivalis, lipopolysaccharide-stimulated cytokine expression, and NF-κB activation. Porphyromonas gingivalis is a periodontal infection that contributes to RA. Active components (like diallyl sulfide) in garlic reduced the expression of NF-κB-dependent genes in mice with UV-B-irradiated skin [80].

In human intestinal epithelial cells, 6-Shogaol, a ginger component, inhibited the PI3K/Akt and NF-κB signaling pathways [81]. Research showed that red ginger extract decreased paw edema in a rat adjuvant arthritis model [82]. Clinical trials showed that ginger therapy dramatically reduced the expression of the *T-bet gene* in rheumatoid arthritis patients. *T-bet* is a transcription factor for T helper (Th1) cells that promotes their proliferation in autoimmune disorders [83].

It is notable that nutrients, foods, and herbal supplements that contain a considerable quantity of fiber, antioxidants and anti-inflammatory mediators can contribute to the mitigation of RA incidence and/or signs and symptoms. On the other hand, a high-sugar diet or drink contributes to the promotion of inflammatory action and the incidence or exacerbation of RA.

### Different diet types to manage RA

Various studies have explored the efficacy of additional therapeutic approaches for RA (Table 6), including fasting as a complementary treatment, the Mediterranean diet, the Cretan Mediterranean diet, vegetarian diet as anti-inflammatory diets, and the utilization of diverse specific foodstuffs in conjunction with standard drug therapy [84].

Fasting, along with adherence to the Mediterranean diet and specifically the Cretan Mediterranean diet, as well as the adoption of anti-inflammatory diets, has demonstrated the potential to positively impact the therapeutic approach toward RA. This is evidenced by the notion that diet can serve as a supplementary component to the conventional drug therapy for the management of RA. Moreover, it has been documented that the implementation of anti-inflammatory diets led to a notable decrease in pain compared with regular diets [85].

**Table 6.** Types of diets for management of RA and their impact on disease symptoms.

Diet types	Diet components									Effects on RA
	Vegetables	Fruits	Oils	Fish and seafood	Dairy products	Eggs	Poultry	Red meat	Grains	
Mediterranean diet	A wide range of raw and prepared vegetables, including legumes.	Varied range of fruits	In the main Cold-pressed extra virgin olive oil	Yes	Yes	Yes	Yes	Yes in small portions	Grains, nuts, seeds	Increase physical function, and enhance quality of life
										Decrease inflammation
										Increase physical function and vitality
										Decrease pain, stiffness, and inflammation
										Decrease pain and inflammation
Vegetarian diet	A wide range of raw and prepared vegetables, including legumes.	Varied range of fruits	Varied range of plant-based oils	Yes or no	Yes or no	Yes or no	No	No	Grains, nuts, seeds	Useful for the treatment of RA
										Decrease inflammation
										Decrease pain and inflammation
Vegan diet	A wide range of raw and prepared vegetables, including legumes.	Varied range of fruits	Varied range of plant-based oils	No	No	No	No	No	Grains, nuts, seeds	Decrease pain and inflammation
Elimination diet	The foods that typically initiate the onset of the ailment must be eliminated from one's dietary intake. Coffee must be excluded.	Trigger foods should be restricted	Varied range of plant-based oils	Yes or no	No	No	Yes or no	No	No Corn, oats and rye	Decrease pain and inflammation
										Decrease inflammation
										Decrease inflammation and the number of tenders

Data taken from [85].

### Genetic effects on rheumatoid arthritis treatment

**Figure 8.**
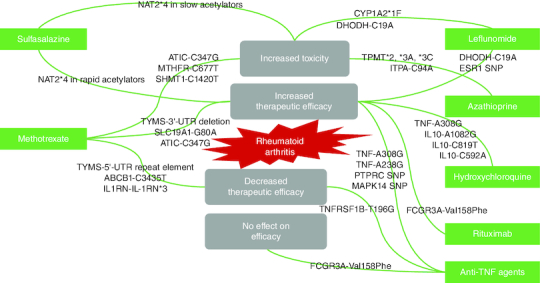
Diagram showing the effect of different genetic variants on rheumatoid arthritis therapeutic outcomes. **(A)** The therapeutic outcome is affected by the existence of different genetic variants. **(B)** Therapeutic efficacy may increase or decrease, toxicity may occur, or no change in efficacy may be seen.

Genetic screening can be introduced in pharmaceutical care services to improve clinical outcomes and enhance the quality of life of RA patients. Certain polymorphic genes may affect, positively or negatively, the therapeutic response or metabolic fate of certain therapeutic molecules (Figure 8).

The inclusion of pharmaceutical care services in the treatment regimen of RA patients can improve the diagnosis and prevention of drug-related issues. Furthermore, it demonstrated a substantial increase in patient adherence and sense of wellbeing [86].

Regarding traditional DMARDs, several SNPs in genes encoding proteins involved in the nucleotide synthesis, drug transport, folate pathway, and cytokine production may be linked to altered efficacy or toxicity of methotrexate (MTX) [87]. The *C3435T SNP* in the *ABCB1* gene, which codes for the MDR1 efflux pump, is involved in drug transport and increases MTX efflux [88]. *ATIC* and *TYMS* are two genes involved in nucleotide synthesis that have been linked to MTX activity. Mutations in these genes alter mRNA stability and enzyme activity, resulting in increased efficacy but high toxicity [89]. Furthermore, an SNP in the *IL1RN gene* has been linked to reduced MTX effectiveness [90].

From the previous data, it is clear that most of the DMARDs and biological agents are positively affected, regarding their clinical efficacy and toxicity, by different genetic polymorphisms. This underlines the importance of applying genetic screening in healthcare settings in order to provide patients with the most suitable therapeutic choice with minimal side effects and maximum clinical efficacy.

### Active & controlled cases how to manage

Over the last few decades, RA care has evolved dramatically, resulting in improved treatment and quality of life for RA patients.

A study showed that in early active RA, the use of tripe therapy sulfasalazine [SSZ]/methotrexate [MTX]/hydroxychloroquine [HCQ] provided highly effective control of disease activity [91]. Furthermore, a clinical study on recently detected RA indicated that the initial combination therapy, including MTX, SSZ and prednisone, or MTX and infliximab, resulted in quicker clinical improvement and slower development of joint damage than the initial monotherapy [92].

Many individuals with controlled RA can retain therapeutic remission. Current therapeutic advice is that glucocorticoids should be discontinued first, followed by a reduction and discontinuation of biologics, and finally, in cases of sustained remission, conventional DMARDs such as methotrexate should be reduced and possibly discontinued. Low disease activity at onset, negative serological testing and a short disease duration after commencing DMARD treatment all contribute to the efficacy of reducing antirheumatic treatments [93].

### Study limitations

Gene therapy approaches are not included in the current review.

## Conclusion

The balance between ideality and reality in pharmacological therapeutic management, dietary intake and lifestyle behaviors such as physical activity, smoking and alcohol use have an impact on the quality of life of patients with established RA. When addressing the management of RA patients, healthcare professionals may need to provide a tailored regimen/counseling for each patient depending on the patient's health state, disease progression, and current diet and lifestyle to achieve the required improvement in quality of life. Fasting as a complementary treatment along with a Mediterranean diet, vegetarian diet, and vegan diet showed promising results in relieving RA symptoms. Moreover, data revealed that diet with low glycemic index, diet reach in fibers, antioxidants and anti-inflammatory mediators beside therapeutic treatment contribute in enhancing RA treatment outcomes. Supplements such as selenium, α-tocopherol, bromelin, turmeric, vitamin C, n-3 fatty acids, and zinc may contribute positively in reliving RA symptoms and enhancing quality of life in RA management. For future perspective, the integration of genetic and epigenetic data, along with the implementation of machine learning algorithms, holds the potential for truly personalized treatments. With the ongoing advancements in genetic and cell-based therapies, the long-awaited cure for rheumatoid arthritis may finally be attainable. Furthermore, the utilization of continuous treatment adaptations, with a focus on managing comorbidities and addressing lifestyle requirements, could also be made possible. These progressive developments in the field of medicine could significantly enhance the quality of life for individuals suffering from RA.
